# Therapeutic Strategies for Abdominal Aortic Aneurysm: A Comprehensive Systematic Review

**DOI:** 10.3390/jcdd12120462

**Published:** 2025-11-27

**Authors:** Egle Kavaliunaite, Joachim Sejr Skovbo Kristensen, Sissel Scheurer, Ida Berg, Jes Sanddal Lindholt, Jane Stubbe

**Affiliations:** 1 Cardiovascular and Renal Research Unit, Institute for Molecular Medicine, University of Southern Denmark, 5230 Odense, Denmark; 2 Department of Cardiac, Thoracic and Vascular Surgery, Odense University Hospital (OUH), 5000 Odense, Denmark; 3 Clinical Institute, University of Southern Denmark, 5000 Odense, Denmark; 4 Clinical Institute, Copenhagen University, 2100 Copenhagen, Denmark

**Keywords:** abdominal aortic aneurysm, drug repurposing, animal models

## Abstract

Background: Abdominal aortic aneurysm (AAA) is a life-threatening condition with no proven pharmacological treatment to halt its progression. While animal models offer insights into pathophysiology and drug response, clinical translation remains limited. Methods: We conducted a systematic review of repurposed drugs, classified by Anatomical Therapeutic Chemical (ATC) codes, tested in animal models for their effects on AAA progression. Following Preferred Reporting Items for Systematic reviews and Meta-Analyses (PRISMA) guidelines and a PROSPERO-registered protocol (CRD42024323430), we screened 14,127 articles and included 144 studies across 13 of the 14 ATC categories. Results: Most drug classes, particularly cardiovascular, metabolic, and immunomodulatory agents—including statins, angiotensin II receptor blockers (ARBs), metformin, and rapamycin—showed a reduced aneurysm diameter. However, high heterogeneity in models, treatment timing, and methodological shortcomings, including a lack of blinding and power calculations, limit translational value. The predominance of positive findings suggests potential publication bias. Conclusions: Nevertheless, drugs effective post-aneurysm initiation may offer the greatest clinical promise. Our findings underscore the need for standardized, high-quality, preclinical research to support future human trials.

## 1. Introduction

An abdominal aortic aneurysm (AAA) is a progressive, permanent dilation of all aortic layers of the abdominal aorta that tends to be asymptomatic and often detected only randomly or through national screening programs [1]. Due to their silent progression, AAAs can develop over many years and may eventually rupture, resulting in a mortality rate of approximately 80% and necessitating emergency surgical intervention [1]. The 2024 European Society for Vascular Surgery (ESVS) guidelines recommend non-surgical management of small, asymptomatic AAAs with regular imaging surveillance, strict blood pressure control, statin therapy, and smoking cessation. These measures aim to slow aneurysm growth and reduce cardiovascular risk, potentially delaying the need for surgery [2]. The current management strategy for diagnosed AAAs involves watchful waiting, with periodic ultrasound surveillance, and surgical treatment is recommended when the aneurysm diameter reaches 5.5 cm in men and 5.0 cm in women [2,3]. This period of watchful waiting presents an opportunity to potentially limit AAA progression through pharmacological means, thus avoiding surgery altogether [4].

Despite extensive research, the exact mechanisms driving AAA development, particularly inflammation and extracellular matrix degradation, remain only partially understood [5]. In response, numerous studies have explored a variety of pharmacological agents, including metformin, which has shown promise in animal models and is currently being evaluated in several human clinical trials [6,7]. Additionally, investigations into vitamins, dietary supplements such as Omega-3 fatty acids, antibiotics, and even stem cells have been conducted to curb AAA expansion [7,8,9].

In light of these developments, drug repurposing has emerged as one of the most promising strategies in the quest for effective medical therapies for AAAs, particularly focusing on repurposed drugs with Anatomical Therapeutic Chemical (ATC) codes [10]. It utilizes existing medications, whose safety profiles are already established, to fast-track the discovery of new treatments. This approach not only saves time and resources but also enhances the potential for clinical application based on pre-existing evidence from various studies [10].

The increasing volume of experimental data from animal studies highlights the need to systematically review these findings for their potential to treat patients with AAAs. Given the ethical and logistical challenges of early-phase clinical trials in AAAs, animal models serve as a vital intermediary, allowing researchers to explore mechanistic pathways and therapeutic efficacy before human application [11,12]. They allow for a detailed examination of the effects of potential treatments on AAA progression, providing foundational knowledge that can inform future clinical trials.

A recent systematic review and meta-analysis by Kristensen et al. [7] examined mainly clinical data on repurposed drugs and dietary supplements in patients with AAAs and reported only small, heterogeneous effects of treatments such as statins and metformin on aneurysm growth, with no convincing reduction in rupture or need for repair. Thus, despite extensive pharmacological research, there is still no medical therapy that can be recommended for routine clinical use. The present review addresses the preclinical testing by systematically mapping repurposed drugs with ATC codes tested in animal AAA models, evaluating their efficacy, study quality, and treatment timing (preventive vs. post-induction), with the aim of identifying novel drug candidates with the strongest experimental support for future clinical trials.

This systematic review aims to provide an up-to-date overview of the pharmacological medications’ ability to halt AAA progression in animal models, evaluating their efficacy, and determining which candidates show the most potential for clinical use. 

## 2. Materials and Methods

### 2.1. Design and Protocol

This systematic review was conducted following the Preferred Reporting Items for Systematic Reviews and Meta-Analyses (PRISMA) guidelines. The review protocol was registered with PROSPERO and is accessible online, CRD42024323430. The last search was performed on 30 January 2025.

### 2.2. Literature Search Strategy

The search strategy was developed in collaboration with a medical research librarian to specifically target studies investigating AAAs in animal models. A comprehensive search was conducted in the following databases: PubMed, Embase (OVID), Web of Science.

The main author and a medical research librarian designed and tested the search string. The search string is set up demanding the two following criteria: (1) abdominal aortic aneurysm, (2) animal study.

Given the exploratory nature of this review, the search strategy prioritized sensitivity over specificity to ensure a broad capture of relevant studies; thus, the search string did not involve treatment keywords.

The search string was as follows:

(Abdominal Aortic Aneurysm* or Abdominal Aorta Aneurysm*) or Abdominal Aortic Aneurysm/and (Animal filter).

Animal filter citation: ISSG Search Filter Resource [Internet]. Glanville J, Lefebvre C, Manson P, Robinson S and Shaw N, editors. York (UK): The InterTASC Information Specialists’ Sub-Group; 2006 [updated 11 February 2022; cited 11 February 2022]. Available from https://sites.google.com/a/york.ac.uk/issg-search-filters-resource/home.

### 2.3. Inclusion and Exclusion Criteria

#### 2.3.1. Inclusion Criteria

Studies were included if they met the following criteria:

1. Preclinical animal studies inducing aortic aneurysms.
2. Investigated pharmaceutical drugs classified under the Anatomical Therapeutic Chemical (ATC) system for their effects on AAA progression, development, or growth.
3. Reported aneurysm size measurements as an outcome to evaluate treatment effectiveness.
4. Included a control group for comparison.
5. Published in a peer-reviewed journal.
6. Written in English.

#### 2.3.2. Exclusion Criteria

Studies were excluded if they met the following criteria:

1. Investigated treatments that do not have an ATC code.
2. Used experimental therapies, including stem cells, nanoparticles, or gene therapy.
3. Focused on dietary supplements, vitamins, or nutrition-based interventions.
4. Were tissue- or cell-based studies (ex vivo or in vitro research).
5. Used genetic modifications unrelated to the AAA model.
6. Studied AAA associated with Marfan syndrome, Ehlers–Danlos syndrome, or other genetic disorders.

### 2.4. Article Selection Process

All identified articles were imported into EndNote Version 20 for reference management and duplicate removal. Further screening and data extraction were facilitated using Covidence, which enabled additional duplicate identification and removal.

**Figure 1:**
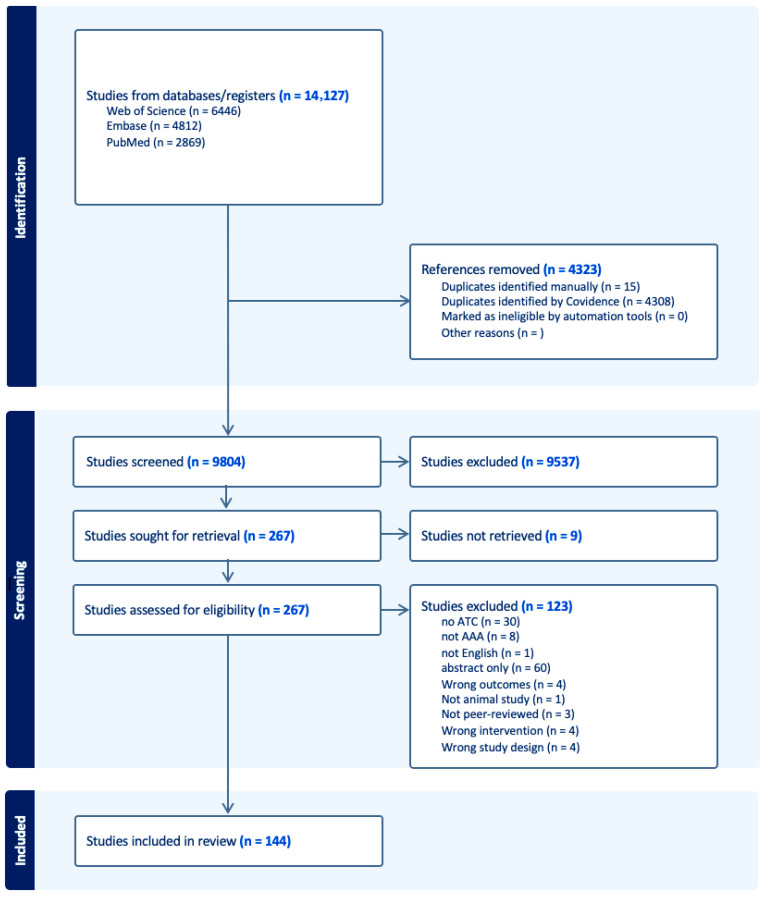
PRISMA flowchart.

Disagreements between reviewers were resolved through discussion, with no senior author intervention required. A PRISMA flowchart (Figure 1) illustrates the article selection process.

### 2.5. Data Extraction

The following study characteristics were recorded:

- General Study Information: Title, authors, publication year, journal.
- Animal Model Details: Species, aneurysm induction method, sample size, study duration.
- Pharmacological Intervention: Drug name, ATC classification, dosage, route of administration.
- Outcome Measures: AAA size changes.
- Extracted data were categorized by ATC drug classifications at the 1st level. Any discrepancies were resolved through discussion and reassessment by the two reviewers.

### 2.6. Risk of Bias Assessment

The risk of bias was assessed using the Systematic Review Centre for Laboratory Animal Experimentation (SYRCLE) risk of bias tool, which is adapted from the Cochrane Collaboration’s risk of bias tool for animal studies.

This tool evaluates ten domains under five categories, including the following:

Selection Bias: Sequence generation, baseline characteristics, allocation concealment.

Performance Bias: Random housing, blinding (caregivers and investigators).

Detection Bias: Random outcome assessment, blinding (outcome assessors).

Attrition Bias: Incomplete outcome data.

Reporting Bias: Selective outcome reporting.

Other Bias: Bias that does not fit into the above categories.

Studies were classified as having a low, high, or medium risk of bias based on SYRCLE’s criteria.

## 3. Results

### 3.1. Study Selection

A total of 14,127 articles were identified. After deduplication and screening of titles and abstracts, 267 full texts were assessed. A total of 144 studies met the inclusion criteria and were included in the final analysis. The selection process is detailed in Figure 1 (PRISMA flow diagram).

### 3.2. Overview of Included Studies

Studies were published between January 1996 and January 2025, primarily using murine and rat models. AAA was induced via porcine pancreatic elastase, Angiotensin-II or calcium chloride. All studies investigated pharmaceutical agents with valid ATC codes, and included control groups.

Outcomes included AAA size changes. The duration ranged from 2 to 84 days, with administration routes including intraperitoneal (IP), oral (per os, PO), subcutaneous (SC), and intravenous (IV), typically administered once daily. Reported randomization, blinding, and power calculation were noted.

Tested drugs spanned 13 of the 14 ATC main classes.

### 3.3. ATC Groups

#### 3.3.1. ATC Group A–Alimentary Tract and Metabolism

A total of 17 studies evaluated pharmacological agents from ATC Group A, primarily focusing on anti-type 2 diabetic and metabolic drugs. There were 4 studies in rats and 13 in mice using different doses, delivery methods, and treatment intervals (Table S1).

Biguanides ATC code: A10BA included a total of 17 studies. An overview of doses, animal models, ways of delivery and frequency, initiation of treatment, and effect of treatment is displayed in Table 1. Four studies included biguanides, mostly focusing on metformin. Across all metformin studies, a consistent reduction in aneurysm progression was observed [13,14,15,16]. Three studies investigated thiazolidinediones (rosiglitazone (A10BG02) and pioglitazone (A10BG03), where both therapies reduced aortic expansion [17,18,19]. Two GLT2 inhibitors, dapagliflozin (A10BK01) and empagliflozin (A10BK03), also attenuated AAA growth [20,21].

The same effects were observed with DPP-4 inhibitors (sitagliptin (A10BH01), alogliptin (A10BH04), teneligliptin (A10BH08)), where all three agents inhibited AAA progression significantly [22,23,24]. This was further supported by the GLP-1 analogs (liraglutide (A10BJ02), exenatide (A10BJ03), where, in all three studies, treatments reduced aneurysm diameter [25,26,27].

Thus, all anti-diabetic therapies led to a reduction in AAA growth. Overall, nine studies reported treatment being initiated after AAAs.

Vitamin D analogs belong to the A group of the ATC code; here, only two studies were included, both reducing AAA growth.

In two studies focused on vitamin D analogs (A11C) [28,29], calcitriol supplementation attenuated AAA development [28,29]. Both studies reported that treatment was started after AAA initiation.

#### 3.3.2. ATC Group B–Blood and Blood Forming Organs

A total of eight studies investigated pharmacological agents classified under ATC Group B, including platelet aggregation inhibitors, factor Xa/IIa inhibitors, proteinase inhibitors, and folic acid.

The most commonly studied class was platelet aggregation inhibitors (four studies), testing clopidogrel (B01AC04) and cilostazol (B01AC23) [30,31,32,33]. Both agents demonstrated significant reductions in aneurysm diameter with no observation of increased ruptures or death due to bleeding. While complement Factor Xa and IIa inhibitors, including rivaroxaban, enoxaparin, fondaparinux, and dabigatran, were evaluated in two studies, all these inhibitors led to a reduction in AAAs [34,35]. Similarly, a proteinase inhibitor, Ulinastatin (B02AB05), also reduced AAA formation [36]. When combining folic acid (vitamin B9, B03BB01) with a calcium channel blocker, only folic acid alone showed beneficial effects on aneurysm progression attenuation [37].

Of the eight included studies, all (100%) reported a reduction in AAA formation, indicating the therapeutic potential of Group B drugs in AAA modulation, though four studies employed pre-treatment protocols that may prime the aorta and prevent or delay the initiation of AAA development. See Table S1.

#### 3.3.3. ATC Group C–Cardiovascular System

A total of 55 studies were classified under ATC Group C, making this the most extensively represented pharmacological group in this review. These studies encompassed a broad range of cardiovascular medications, including blood pressure-modulating agents such as Angiotensin receptor blockers (ARBs), angiotensin converting enzyme (ACE) inhibitors, calcium channel blockers, cardiac glycosides, and beta-blockers, but also cholesterol-lowering drugs like statins and fibrates, and other related compounds.

ARBs (ATC code C09CA), directly affecting AT1 receptor signaling, were featured in 13 studies, highlighting their prominent role in AAA modulation. Compounds such as valsartan, losartan, telmisartan, candesartan, and irbesartan were associated with reductions in aneurysm diameter. Eleven studies reported a reduction in AAA size findings [38,39,40,41,42,43,44,45,46,47,48], while two studies did not observe any effects [49,50]. Similarly, ACE inhibitors (C09AA), preventing angiotensin II formation, including perindopril, lisinopril, imidapril, and benazepril, were evaluated in nine studies, all of which reported protective outcomes [42,44,51,52,53,54,55,56]. In addition, blood pressure-lowering calcium channel blockers (C08CA, C08DB) were examined in six studies, all of which demonstrated beneficial effects. Amlodipine, nifedipine, diltiazem, and azelnidipine were all associated with a significant reduction in aortic diameter [37,57,58,59,60,61]. Mineralocorticoid receptor antagonists like spironolactone (C03DA01) [62], and the vasopressin antagonist tolvaptan (C03XA01) [63], also showed beneficial effects preventing AAA growth. Similarly, renin inhibitors (C09XA02) were effective in limiting AAA growth [64,65], as was hydralazine (C02DB02) [43,66].

Cardiac glycosides such as digoxin (C01AA05), known to prevent T helper 17 cell differentiation and modulate hypoxia-inducible Factor-1alpha, were studied by two independent groups, both showing protection against AAA development [67,68], while beta-blockers (C07AA05), specifically propranolol, were evaluated in four studies with mixed findings. Simpson et al. and Ricci et al. documented a reduction in AAA expansion, whereas Slaiby et al. and Park et al. observed minimal or no effect [52,69,70,71]. These results also included mixed AAA models and species.

The cholesterol-lowering statins (C10AA), used as standard care in patients with AAA, were studied in 16 studies. Statins, including simvastatin, atorvastatin, rosuvastatin, fluvastatin, and pravastatin, were investigated. Of these, 14 reported significant protective effects against AAA [46,50,72,73,74,75,76,77,78,79,80,81,82,83], while 2 found no beneficial effects [84,85]. Fibrates used to manage the cholesterol-lowering effect through the activation of proliferator-activated receptor alpha (PPAR alpha), such as fenofibrate and pemafibrate (C10AB05, C10AB12), were evaluated by three studies. All studies reported reduced AAA progression [17,86,87]. The lipid-lowering Probucol (C10AX02) also reduced AAA diameter by effectively reducing aortic wall degeneration [88,89]. Finally, the old cholesterol-lowering Niacin (vitamin B3, C10AD02) demonstrated attenuation of aneurysm growth [90].

In summary, of the 55 studies reviewed under ATC Group C, 50 reported beneficial effects in preventing AAA expansion. Notably, 29 of these studies applied treatment before AAA induction. See Table S1.

#### 3.3.4. ATC Group D–Dermatologicals

Only one study investigated an agent from ATC Group D. This study examined the effects of all-trans retinoic acid (D10AD02), a retinoid typically used for dermatological conditions [91]. Treatment was administered prior to aneurysm induction. The study reported significant attenuation of aneurysm progression, including reductions in maximum aortic diameter and occurrence of AAA. See Table S1.

#### 3.3.5. ATC Group G–Genito-Urinary System and Sex Hormones

A total of four studies investigated pharmacological agents from ATC Group G. These included androgen receptor antagonists, alpha-adrenoreceptor antagonists (G04CA02], and a selective estrogen receptor modulator [92,93,94,95]. All four studies reported a positive effect in reducing the diameter of AAA, though one study initiated treatment before aneurysm induction [93]. See Table S1.

#### 3.3.6. ATC Group H–Systemic Hormonal Preparations

Only one study investigated a drug from ATC Group H, specifically assessing the effects of methylprednisolone (H02AB04), a glucocorticoid, on experimental AAA formation in rats. This study also evaluated cyclosporine (L04AD01), an immunosuppressant classified under ATC Group L, and is therefore discussed in both sections [96]. Treatment began after the induction of the aneurysm. The study found that methylprednisolone reduced aneurysm diameter significantly. See Table S1.

#### 3.3.7. ATC Group J–Anti-Infective for Systemic Use

A total of 14 studies were categorized under ATC Group J, which includes systemic anti-infectives. Within this group, the primary focus was on antibiotics, particularly tetracyclines. Ten studies investigated the effects of tetracyclines (J01AA), primarily doxycycline and minocycline, on AAA progression. Nine studies found that tetracycline reduced AAA growth [50,97,98,99,100,101,102,103,104,105], while one study found no change in AAA diameter [106]. The remaining four studies assessed other classes of antibiotics, including macrolides, beta-lactams, or combinations [50,107,108,109]. Three studies reported a positive effect of attenuating AAA growth [107,108,109], while one study did not report attenuating AAA growth [50].

In total, 10 of the 14 studies (71%) concluded with positive outcomes, reinforcing the potential of anti-infectives in non-traditional, anti-inflammatory roles. A total of 11 studies applied therapeutic treatment, while 3 studies used preventive protocols. See Table S1.

#### 3.3.8. ATC Group L–Antineoplastic and Immunomodulating Agents

ATC Group L encompasses antineoplastic, endocrine therapy, and immunomodulating agents, and included a total of 22 studies. A total of 10 studies evaluated antineoplastic agents such as imatinib, rapamycin, and others. All of the studies showed to be protective against AAA growth [72,110,111,112,113,114,115,116,117,118]. Also, immunosuppressive therapies beyond corticosteroids were explored in 10 studies. The most frequent agent was interleukin 1 receptor antagonist, Anakinra. In all studies, treatment prevented AAA expansion [96,119,120,121,122,123,124,125,126,127].

The final two studies in this ATC group examined endocrine therapy, including the non-steroidal anti-androgen flutamide and the estrogen receptor modulator tamoxifen. Both studies showed a positive effect of attenuating AAA growth [92,128]. In total, 22 studies reported beneficial effects on AAA size. Three of the studies (14%) initiated treatment before AAA induction [50,98,99]. See Table S1.

#### 3.3.9. ATC Group M–Musculoskeletal System

ATC Group M, which comprises medications targeting the musculoskeletal system, was represented across nine studies involving anti-inflammatory agents, anti-gout medications, and bisphosphonates.

NSAIDs, particularly indomethacin, were investigated in two studies [129,130]. Both Holmes et al. and Miralles et al. reported a significant reduction in AAA progression in rat models. Additionally, celecoxib, a COX-2 selective inhibitor, demonstrated efficacy in attenuating AAA growth in an angiotensin II-infused mouse model, but no change in rupture rate [131]. Colchicine, a classic anti-gout agent, was assessed in four separate studies. While Phie et al. reported no significant impact on aneurysm development [132], three more recent investigations showed promising results [133,134,135]. These studies suggested that colchicine inhibited the development of AAA. Finally, Zoledronate, a bisphosphonate, was also evaluated for its effect on AAA progression. Tsai et al. found that it attenuated angiotensin II-induced aneurysm formation in mice [136]. The same agent, risedronate, was included as part of a combination therapy in the study by Nakahara et al. [46] and showed positive results of attenuating AAA growth [46].

Overall, eight of the nine studies showed a beneficial effect on AAA outcomes. Seven studies (78%) reported that medical treatments were initiated after aneurysm induction. See Table S1.

#### 3.3.10. ATC Group N–Nervous System

A total of 11 studies were categorized under ATC Group N, which includes drugs that primarily target the nervous system. This group encompassed a diverse range of pharmacological agents, including antiepileptics, hypnotics and sedatives, psychoanaleptics, and others. Hypnotics and sedatives were the largest group, including five studies, with melatonin being in three studies [137,138,139,140,141]. All studies diminished AAA growth. In addition, antiepileptics were studied in two studies. Both studies used topiramate and reported a positive effect in attenuating AAA growth [142,143]. Also, the psychoanaleptics group was represented by vinpocetine, which showed a positive effect in attenuating AAA growth [144]. Lastly, other nervous system agents such as edaravone and disulfiram were featured in three additional studies [145,146,147]. All showed a positive effect of treatment to attenuate AAA development.

Overall, 11 out of 11 studies reported positive effects on AAA progression. Pre-treatment was initiated in five studies (45%). See Table S1.

#### 3.3.11. ATC Group P–Antiparasitic Products

This group, representing antiparasitic agents, was investigated in two studies exploring the therapeutic potential of disulfiram and chloroquine in experimental models. Disulfiram, a sulfur-containing compound, effectively reduced AAA progression in angiotensin II-induced mice [148], while chloroquine, an aminoquinoline anti-malarial agent, did not suppress AAA growth [149].

Both studies reported treatment being started after the initiation of AAA. See Table S1.

#### 3.3.12. ATC Group R–Respiratory System

Five studies were identified under ATC Group R, which includes medications primarily indicated for respiratory conditions such as asthma. A selective beta-2-adrenoreceptor agonist (R03AC13), Formoterol, was evaluated in a murine model, showing attenuation of AAA progression [150]. Montelukast, a leukotriene receptor antagonist (R03DC03), was explored in four studies, all reporting positive outcomes of attenuating AAA growth [151,152,153,154]. All five studies demonstrated positive findings, with observed effects including reduced AAA size. One [154] of the five studies (20%) initiated treatment before AAA operation. See Table S1.

#### 3.3.13. ATC Group S–Sensory Organs

ATC Group S is represented by a single study investigating the role of an anti-neovascularization agent. In this study, the effects of inhibiting Yes-associated protein 1 (YAP1) on AAA formation in mice significantly reduced AAA formation, and the treatment was initiated prior to AAA induction [155]. See Table S1.

#### 3.3.14. Risk of Bias Assessment Results

SYRCLE risk of bias tool was used to assess the risk of bias. Each article was assessed according to five criteria/domains: selection bias, performance bias, detection bias, attrition bias, reporting bias, and other bias. Overall, only 7 studies were deemed to be of low risk of bias, and 67 studies were deemed at medium risk, while the majority of the studies (69 studies) were deemed at high risk of bias. The distribution of overall SYRCLE risk of bias across ATC groups is shown in Table 1. The full table is found in the Supplementary Materials, Table S2. 

**Table 1:** Summary of Overall SYRCLE Risk of Bias Stratified by ATC Group.

ATC Group	Main Category	Total	Low Risk n (%)	Medium Risk n (%)	High Risk n (%)
**A**	Alimentary tract and metabolism	17	0 (0.0%)	9 (52.9%)	8 (47.1%)
**B**	Blood and blood-forming organs	8	0 (0.0%)	4 (50.0%)	4 (50.0%)
**C**	Cardiovascular system	50	3 (6.0%)	19 (38.0%)	28 (56.0%)
**D**	Dermatologicals	1	0 (0.0%)	1 (100.0%)	0 (0.0%)
**G**	Genito-urinary system and sex hormones	3	0 (0.0%)	3 (100.0%)	0 (0.0%)
**H**	Systemic hormonal preparations	1	0 (0.0%)	1 (100.0%)	0 (0.0%)
**J**	Anti-infectives for systemic use	13	0 (0.0%)	9 (69.2%)	4 (30.8%)
**L**	Antineoplastic and immunomodulating agents	22	1 (4.5%)	9 (40.9%)	12 (54.5%)
**M**	Musculoskeletal system	8	1 (12.5%)	2 (25.0%)	5 (62.5%)
**N**	Nervous system	12	2 (16.7%)	5 (41.7%)	5 (41.7%)
**P**	Antiparasitic products	2	0 (0.0%)	1 (50.0%)	1 (50.0%)
**R**	Respiratory system	5	0 (0.0%)	3 (60.0%)	2 (40.0%)
**S**	Sensory organs	1	0 (0.0%)	1 (100.0%)	0 (0.0%)

## 4. Discussion

This systematic review presents a comprehensive evaluation of repurposed pharmaceutical agents with valid ATC classifications tested in animal models of AAA. A total of 144 studies were included, spanning 13 of the 14 ATC main categories. Across these diverse drug classes, the majority demonstrated a reduction in AAA progression based on AAA diameter.

Moreover, translating these findings to clinical practice remains challenging. In humans, there is a large variability, influenced by comorbidities, timing of treatment initiation (after AAA is developed), and whether treatment is for an extended period of years-long management, with long-term outcomes and side effects, which are difficult to replicate in animal models often using inbred strains, housed together “life-style” for a short period of time (14–28 days for most AAA models). For instance, while agents like statins and ARBs show clear benefits preclinically, human data are more mixed and often limited by study design [49,81,156,157].

The preclinical drug testing summarized in this review shows predominantly positive effects on AAA progression. However, when these findings are considered alongside the generally high risk of bias in the animal studies, the apparent efficacy must be interpreted cautiously. Our observations for statins and metformin complement the meta-analysis by Kristensen et al. [7], which evaluated pharmacological treatments in human studies and found only modest and uncertain benefits. In line with Kristensen et al., we also observe beneficial effects in preclinical models, but most studies initiated treatment before or shortly after AAA induction and were of only medium to low methodological quality with respect to randomization and blinded outcome assessment.

Among the largest and most consistently effective drug groups in this review were those from ATC Group C (Cardiovascular system), which comprised the largest number of studies (n = 55). Agents such as ARBs [39,46,48], ACE inhibitors [54,56], statins [74,75], and calcium channel blockers [59,61] were all associated with reductions in aortic expansion, and often independently of their hemodynamic effects [72,78]. Cardiovascular agents dominate this review, reflecting a shared recognition of their plausible mechanistic relevance to AAA pathophysiology, particularly through the modulation of hemodynamics, inflammation, and matrix degradation [61,85]. However, while preclinical studies report consistent protective effects across a wide spectrum of cardiovascular drugs, models used, and in different species, clinical findings are far more cautious [7]. Three clinical trials evaluated the effects of anti-hypertensive medications on AAA progression. Propranolol was tested in two trials [158,159], while a third trial compared amlodipine and perindopril against a common control group [160]. None of these medications significantly reduced AAA growth, rupture, or the need for surgery compared to the placebo. Three additional trials tested novel pharmacological approaches to slow AAA progression. The AORTA trial assessed three doses of the mast cell inhibitor pemirolast [161], the FAME-2 trial evaluated fenofibrate’s effect on AAA biomarkers and growth [162], and the TicAAA trial examined ticagrelor, an antiplatelet agent, for its impact on AAA expansion [163]. Despite promising preclinical evidence, none of the interventions significantly reduced AAA growth rate or related clinical events over 12 months. In patients, it has been reported that statins showed small but statistically significant reductions in growth rate in the human meta-analysis, though these effects were highly heterogeneous and derived from studies with methodological limitations [7]. Although the evidence is limited, statins are today recommended for all patients diagnosed with AAA, according to the ESVS 2024 clinical guidelines [2]. A forthcoming publication in *Circulation*, based on data from the VIVA and DANCAVAS studies, demonstrates a clear dose-dependent association between statin therapy and reduced AAA growth, surgical intervention, rupture, and mortality [164].

In ATC Group A (Alimentary tract and metabolism), preclinical studies highlighted a diverse set of antidiabetic agents, including metformin, SGLT2 inhibitors, DPP-4 inhibitors, and GLP-1 receptor agonists, all of which demonstrated anti-aneurysmal effects [13,21,23,25]. These agents may act through shared anti-inflammatory, antioxidant, or metabolic pathways, and their efficacy is often extended beyond glucose regulation [13,15,16]. The review by Kristensen et al. [7] confirmed that metformin protected against AAA growth significantly, but the observed dataset displayed a large heterogeneous effect in the human cohorts [7]. Our data suggested that other metabolic agents, such as SGLT2 inhibitors, should be tested in humans as their potential from preclinical studies shows very promising and uniform results.

ATC Group L (Antineoplastic and immunomodulating agents) also emerged as a robust area of preclinical activity, including drugs such as rapamycin, anakinra, and cyclosporine [96,112,122]. These agents target inflammatory cascades, immune cell activation, and matrix remodeling, known to be central processes in AAA development [112,122]. Despite the volume and consistency of animal data, this group is not well characterized in patients with AAA [122] and could be explored.

We find that, in preclinical studies, glucocorticoids protect against AAA expansion [17,86,87] via its immunosuppressant effects. Regarding glucocorticoids, there have been some discussions about whether its immunosuppressive action would prevent AAA growth, but it has also been suggested that there is a potential increase rupture rates in humans [165]. A retrospective cohort study by Tajima et al. evaluated glucocorticoid use in patients with AAA but found a non-significant trend toward increased growth [165]. Likewise, oral glucocorticoids were included in the meta-analyses by Kristensen et al., showing no beneficial effects but substantial heterogeneity [7]. Another immunosuppressant, anakinra, an IL1beta neutralizing antibody, was quite effective in the preclinical studies [122]. Notably, no human studies have yet assessed agents such as rapamycin or anakinra dampening IL-1beta signaling, yet a review by Millar et al. [166] highlights the multifaceted and central role of IL-1beta signaling in mediating aortic wall inflammation and the progression of AAA, underscoring a translational blind spot for mechanistically promising therapies.

ATC Group B (Blood and blood forming organs), including antiplatelet agents and anticoagulants, were commonly studied in the meta-analysis by Kristensen et al. [7], as it is in this review. In preclinical models, agents like clopidogrel and rivaroxaban demonstrated reductions in AAA growth and inflammation [32,34]. However, human evidence remains inconclusive. Kristensen et al. [7] included several large cohort studies and internal data from DANCAVAS and VIVA screening trials [167,168] examining platelet inhibitors and oral anticoagulants, but found no significant association with reduced aneurysm growth rate. These divergent results may reflect differences in treatment timing, patient comorbidities, and the indirect effects of these medications in real-world heterogeneous populations.

Several ATC groups that are minimally explored in human AAA research, such as Group N (nervous system), Group M (musculoskeletal system), and Group R (respiratory system), were represented in this preclinical studies review. Agents such as melatonin, colchicine [133,138] and montelukast [153] showed promising effects in preventing aneurysm progression in rodent AAA models. These findings highlight the exploratory strength of preclinical research and may reveal untapped therapeutic opportunities deserving of further mechanistic and translational studies.

It is essential to consider the quality of the included studies. The risk of bias assessment using the SYRCLE tool revealed substantial variability, with only seven studies classified as low risk [74,78,87,120,132,139,142], while the majority were rated as medium risk (n = 67) or high risk (n = 69). Common issues included poor reporting of randomization, blinding, and power calculation of meaningful sample size. These are all factors known to exaggerate effect sizes and limit reproducibility [169].

A notable finding of this review is that most tested drugs reduced AAA progression. Together with the SYRCLE assessment, this high proportion of positive results raises concerns about methodological limitations and publication bias. Poor reporting of randomization and allocation concealment, limited blinding, lack of power calculations, and incomplete reporting of exclusions all increase the risk of inflated treatment effects and limit reproducibility [169]. Thus, the predominance of favorable findings may reflect not only true therapeutic effects but also selective reporting and suboptimal study design. Estimates suggest that one in six animal studies remains unpublished [170], which could skew the literature toward overly optimistic conclusions. Methodological shortcomings further compound these concerns. Many studies failed to clearly report key elements such as randomization, allocation concealment, blinding, or sample size calculations, which have been standard practices in clinical research for decades [169]. As demonstrated by Hirst et al. [169], omitting these design features in animal studies can lead to inflated effect sizes across various disease models [169], ultimately hindering the identification of the most consistently effective drugs for human trials.

These issues could at least partially explain why many promising preclinical findings fail to translate into clinical benefit. Preventive treatment models, used in a substantial portion of studies, may not reflect real-world clinical settings where AAAs are typically diagnosed post-formation. Therefore, drugs demonstrating efficacy in post-induction models should be prioritized for clinical translation.

The preclinical drug testing results of repurposed drugs for AAA are largely positive, whereas clinical trials have so far shown little or no effect on aneurysm growth or events. In our SYRCLE assessment, only seven studies were judged to be at an overall low risk of bias; these studies mainly evaluated lipid-lowering drugs, IL-1beta pathway blockade, and a few targeting the nervous system and musculoskeletal agents, and generally reported a benefit, while the only low-risk colchicine study was neutral.

Considering both effect and study quality, the most realistic candidates for translation are lipid-lowering agents (statins and fibrates) and RAAS blockers within ATC Group C, metformin and other antidiabetic drugs within Group A, and selected immunomodulators such as IL-1 beta pathway blockade and mTOR inhibition within Group L. Other drugs such as colchicine, melatonin, and montelukast remain promising but exploratory, and should first be confirmed in better-designed animal models. Future preclinical work should prioritize post-induction, randomized, blinded, and adequately powered studies with standardized imaging-based measurements of AAA diameter to better inform human trials. As human trials often build on preclinical data, it is crucial that only well-designed, unbiased animal studies inform clinical decision-making [169]. Studies with substantial methodological flaws should not form the foundation for human trials.

## 5. Conclusions

This systematic review underscores the substantial potential of drug repurposing in AAA, with evidence spanning a broad pharmacological spectrum in preclinical studies. While many findings await clinical validation, the consistency of effects across key drug classes, combined with emerging insights from underexplored agents, presents a compelling case for translational research. Systematic prioritization improved preclinical rigor, and thoughtful clinical trial design will be crucial to realizing the promise of medical therapy for AAA.

## Data Availability

The raw data supporting the conclusions of this article will be made available by the authors on request.

## Supplementary Materials

The following supporting information can be downloaded at https://www.mdpi.com/article/10.3390/jcdd12120462/s1, Table S1: Summary of Studies Investigating Repurposed Pharmacological Agents in Animal Models of Abdominal Aortic Aneurysm (AAA), Categorized by ATC Code. Summary of Studies Investigating Repurposed Pharmacological Agents in Animal Models of Abdominal Aortic Aneurysm (AAA), Categorized by ATC Code; Table S2: Risk of bias table.
