# Supplementary material for: Therapeutic Strategies for Abdominal Aortic Aneurysm: A Comprehensive Systematic Review
Source: J Cardiovasc Dev Dis. 2025 Nov 27;12(12):462. doi: 10.3390/jcdd12120462 (PMC12734017; doi:10.3390/jcdd12120462)
Supplement: Supplementary file 1 [file jcdd-12-00462-s001.zip › jcdd-3993663-supplementary.pdf]

Table S1. Summary of Studies Investigating Repurposed Pharmacological Agents in Animal Models of Abdominal Aortic Aneurysm (AAA), Categorized by ATC Code. Summary of Studies Investigating Repurposed Pharmacological Agents in Animal Models of Abdominal Aortic Aneurysm (AAA), Categorized by ATC Code.

Study ID	ATC code	Medication Group	Type of animal	AAA model	dose (units)	medication administration frequency	treatment adminstration route	relative AAA changes comparing to controls	Treatment Started
He 2021	A10BA02	Antidiabetics, Biguanides	rat	porcine perfusion elastase (PPE)	100 mg/kg/day	everyday	gavage	yes	Treatment started before the initiation of AAA
Kunath 2021	A10BA02	Antidiabetics, Biguanides	mouse	Angiotensin II (Ang II)	100 mg/kg/d	everyday	with water	yes	Treatment started before the initiation of AAA
Wang 2019	A10BA02	Antidiabetics, Biguanides	mouse	Angiotensin II (Ang II)	100 mg/kg/d	everyday	with water	yes	Treatment started after the initiation of AAA
Xu 2023	A10BA02	Antidiabetics, Biguanides	mouse	porcine perfusion elastase (PPE)	250 mg/kg	everyday	gavage	no	Treatment started after the initiation of AAA
Bao 2014	A10BH04	Antidiabetics, DPP-4 inhibitors	rat	porcine perfusion elastase (PPE); calcium choride	1 mg/kg and 3 mg/kg	everyday	gavage	yes	Treatment started before the initiation of AAA
Lu 2015	A10BH01	Antidiabetics, DPP-4 inhibitors	mouse	Angiotensin II (Ang II)	2.5, 5 or 10 mg/kg/day	everyday	gavage	yes	Treatment started after the initiation of AAA
Takahara 2018	A10BH08	Antidiabetics, DPP-4 inhibitors	mouse	Angiotensin II (Ang II)	30 mg/kg/day	everyday	with water	yes	Treatment started before the initiation of AAA

Yu 2016	A10BJ03	Antidiabetics, GLP-1 receptor agonists	rat	porcine perfusion elastase (PPE); calcium choride	10 µg/kg/day or 100 µg/kg/day	everyday	Subcutaneous injection	yes	Treatment started after the initiation of AAA
Zhao 2024	A10BJ02	Antidiabetics, GLP-1 receptor agonists	mouse	porcine perfusion elastase (PPE); calcium choride	300 µg/kg	everyday	Subcutaneous injection	yes	Treatment started after the initiation of AAA
Kohashi 2016	A10BJ	Antidiabetics, GLP-1 receptor agonists	mouse	Angiotensin II (Ang II)	2.16 nmol/kg/day	everyday	with water	no	Treatment started after the initiation of AAA
Liu 2022	A10BK01	Antidiabetics, SGLT2 inhibitors	mouse	porcine perfusion elastase (PPE)	1 or 5 mg/kg	everyday	gavage	yes	Treatment started before the initiation of AAA
Ortega 2019	A10BK03	Antidiabetics, SGLT2 inhibitors	mouse	Angiotensin II (Ang II)	1 mg/kg per day or 3 mg/kg per day	everyday	gavage	yes	Treatment started after the initiation of AAA
Jones 2009	A10BG02	Antidiabetics, Thiazolidinediones	mouse	Angiotensin II (Ang II)	10 mg/kg/d	everyday	with water	yes	Treatment started before the initiation of AAA
Wang 2018	A10BG02	Antidiabetics, Thiazolidinediones	mouse	Angiotensin II (Ang II)	3mg/kg/day	everyday	gavage	yes	Treatment started before the initiation of AAA
Golledge 2010	A10BG03	Antidiabetics, Thiazolidinediones	mouse	Angiotensin II (Ang II)	Pioglitazone: 50 mg/kg	everyday	with water	yes	Treatment started before the initiation of AAA

Jreije 2022	A11CC04	Vitamins, Vitamin D and analogues	mouse	Angiotensin II (Ang II); other	0.1 µg/Kg	every 3 days	intraperitoneal injection	yes	Treatment started after the initiation of AAA
Martorell 2016	A11CC04	Vitamins, Vitamin D and analogues	mouse	Angiotensin II (Ang II)	0.1 µg/kg or 1 µg/kg	every 2 days	gavage	yes	Treatment started after the initiation of AAA
Ding 2021	B01AF01	Blood and blood forming organs, Antithrombotic agents	mouse	Angiotensin II (Ang II)	15 mg/kg/d or 5 mg/kg/d	everyday	gavage	yes	Treatment started after the initiation of AAA
Moran 2017	B01AB05, B01AX05 or B01AE07	Blood and blood forming organs, Antithrombotic agents	mouse	Angiotensin II (Ang II)	enoxaparin 2mg/kg, fondaparinux 300µg/kg or dabigatran intervention chow 7.5mg DE/gram chow	every 2 days	with food; other	yes	Treatment started after the initiation of AAA
Huang 2022	B03BB01	Blood and blood forming organs, Folic acid and derivatives	mouse	Angiotensin II (Ang II)	15 mg/kg	everyday	with food	yes	Treatment started before the initiation of AAA
Liu 2012	B01AC04	Blood and blood forming organs, Platelet	mouse	Angiotensin II (Ang II)	30 mg/kg	everyday	intraperitoneal injection	yes	Treatment started before the initiation of AAA

		aggregation inhibitors							
Owens 2015	B01AC06 or B01AC04	Blood and blood forming organs, Platelet aggregation inhibitors	mouse	Angiotensin II (Ang II)	50 mg/kg clop and 30 mg/L asa	everyday	with food; with water	no	Treatment started after the initiation of AAA
Zhang 2011	B01AC23	Blood and blood forming organs, Platelet aggregation inhibitors	rat	porcine perfusion elastase (PPE)	100mg/kg/da y	everyday	gavage	yes	Treatment started before the initiation of AAA
Umebaya shi 2018	B01AC23	Blood and blood forming organs, Platelet aggregation inhibitors	mouse	Angiotensin II (Ang II)	cilostazol-containing (0.1% wt/wt) diet	everyday	with food	yes	Treatment started before the initiation of AAA
Li 2020	B02AB05	Blood and blood forming organs, Proteinase inhibitors	mouse	porcine perfusion elastase (PPE)	50,000 U/kg	everyday	intraperitone al injection	yes	Treatment started after the initiation of AAA
Alsac 2011	C09AA04	Antihypertens ives, ACE inhibitors	rat	other	3 mg/kg	everyday	with food	yes	Treatment started after the initiation of AAA
Takai 2013	C09AA16	Antihypertens ives, ACE inhibitors	mouse	other	10 mg/kg	everyday	gavage	yes	Treatment started before the initiation of AAA

Xiong 2014	C09AA04	Antihypertensives, ACE inhibitors	rat	porcine perfusion elastase (PPE); calcium choride	3 mg/kg	everyday	with food	yes	Treatment started after the initiation of AAA
Fu 2011	C09AA07	Antihypertensives, ACE inhibitors	rabbit	porcine perfusion elastase (PPE)	3 mg/kg	everyday	gavage	yes	Treatment started before the initiation of AAA
Hao 2018	C09AA	Antihypertensives, ACE inhibitors	mouse	Angiotensin II (Ang II)	1*10^9 pfu	other	intravenous injection	yes	Treatment started after the initiation of AAA
Liao 2001	C09AA01, C09AA03, C09AA02, C09CA01	Antihypertensives, ACE inhibitors / Antihypertensives, Angiotensin II receptor blockers (ARBs)	rat	porcine perfusion elastase (PPE)	4 to 6 mg/kg	everyday	with water	yes	Treatment started after the initiation of AAA
Park 2019	C09AA05, C07AG02	Antihypertensives, ACE inhibitors / Beta blocking agents, Alpha and beta blockers	mouse	Angiotensin II (Ang II)	Ramipril 4.2mg/30g or carvedilol 3.125 mg/30g	everyday	gavage	yes	Treatment started after the initiation of AAA
Daugherty 2001	C09CA01	Antihypertensives, Angiotensin II receptor blockers (ARBs)	mouse	Angiotensin II (Ang II)	30 mg/kg	everyday	Subcutaneous injection	yes	Treatment started after the initiation of AAA

Fujiwara 2008	C09CA03	Antihypertensives, Angiotensin II receptor blockers (ARBs)	rat	porcine perfusion elastase (PPE)	1 mg/kg	everyday	intramuscular injection	yes	Treatment started after the initiation of AAA
Hall 2021	C09CA01	Antihypertensives, Angiotensin II receptor blockers (ARBs)	mouse	calcium choride	30 mg/kg	everyday	Subcutaneous minipump	yes	Treatment started after the initiation of AAA
Hosokawa 2010	C09CA08	Antihypertensives, Angiotensin II receptor blockers (ARBs)	hamster	porcine perfusion elastase (PPE)	5 mg/kg	everyday	gavage	yes	Treatment started after the initiation of AAA
Karasaki 2023	C09CA08	Antihypertensives, Angiotensin II receptor blockers (ARBs)	mouse	calcium choride	20mg/kg	everyday	gavage	no	Treatment started before the initiation of AAA
Kaschina 2008	C09CA07, C02DB02	Antihypertensives, Angiotensin II receptor blockers (ARBs)	rat	porcine perfusion elastase (PPE)	telmisartan 0.5 mg/kg or hydralazine 15mg/kg	everyday	intraperitoneal injection; with water	yes	Treatment started after the initiation of AAA
NguyenTLL 2024	C09CA01	Antihypertensives, Angiotensin II receptor	mouse	Angiotensin II (Ang II)	30 mg/kg	every 2 days	gavage	yes	Treatment started after the initiation of AAA

		blockers (ARBs)							
Xuan 2018	C09CA07	Antihypertensives, Angiotensin II receptor blockers (ARBs)	mouse	porcine perfusion elastase (PPE)	10 mg/kg	everyday	with food	yes	Treatment started before the initiation of AAA
Inoue 2009	C09CA06, C09AA03	Antihypertensives, Angiotensin II receptor blockers (ARBs) / Antihypertensives, ACE inhibitors	mouse	Angiotensin II (Ang II)	candesartan (30 mg/kg), lisinopril (60 mg/kg)	everyday	other	yes	Treatment started after the initiation of AAA
AlSiraj 2021	C09CA03, C09DX04	Antihypertensives, Angiotensin II receptor blockers (ARBs) / Antihypertensives, ARBs in combination	mouse	Angiotensin II (Ang II)	sacubitril 1, 6, or 9 mg/kg, valsartan 0.3, 0.5, 1, 6, or 20 mg/kg or the combination thereof (1 and 0.3 or 9 or 0.5 mg/kg of sacubitril and valsartan	everyday	Subcutaneous minipump	yes	Treatment started before the initiation of AAA
Iida 2012	C09CA04, C09CA07, C10AA04, C02KX01	Antihypertensives, Angiotensin II receptor blockers	mouse	porcine perfusion elastase (PPE);	Irbesartan (50 mg/kg), telmisartan (10 mg/kg), fluvastatin	everyday	with food; with water	no	Treatment started before the initiation of AAA

		(ARBs) / Lipid modifying agents, Statins (HMG CoA reductase inhibitors) / Antihypertensives, Endothelin receptor antagonists		Angiotensin II (Ang II)	(40 mg/kg), bosentan (100 mg/kg), doxycycline (100 mg/kg)				
Wang 2023	C02DB02	Antihypertensives, Hydrazinophthalazine derivatives	mouse	Angiotensin II (Ang II)	24 mg/kg	everyday	with water	yes	Treatment started before the initiation of AAA
Miyake 2017	C09XA02	Antihypertensives, Renin inhibitors	rabbit	porcine perfusion elastase (PPE)	10 mg/kg or 50 mg/kg	everyday	Subcutaneous minipump	yes	Treatment started before the initiation of AAA
Seto 2014	C09XA02	Antihypertensives, Renin inhibitors	mouse	Angiotensin II (Ang II)	10 mg/kg and 50 mg/kg	everyday	Subcutaneous minipump	yes	Treatment started after the initiation of AAA
Simpson 1970	C07AA05	Beta blocking agents, Non-selective	turkey	other	34mg/kg	everyday	with food	yes	Treatment started after the initiation of AAA
Ricci 1996	C07AA05	Beta blocking agents, Non-selective	rat	porcine perfusion elastase (PPE)	10 mg/kg and 30 mg/kg	everyday	with food	yes	Treatment started after the initiation of AAA
Slaiby 1994	C07AA05	Beta blocking agents, Non-selective	rat	porcine perfusion	10 mg/kg or 30 mg/kg	everyday	Subcutaneous injection	yes	Treatment started after the initiation of AAA

				elastase (PPE)					
Chen 2013	C08CA01	Calcium channel blockers, Dihydropyridines	mouse	Angiotensin II (Ang II)	5 mg/kg	everyday	intravenous injection	yes	Treatment started after the initiation of AAA
Huang 2022	C08CA05	Calcium channel blockers, Dihydropyridines	mouse	Angiotensin II (Ang II)	Nifedipine (1.5, 5 or 20 mg/kg/day)	everyday	with food	yes	Treatment started before the initiation of AAA
Miao 2015	C08CA05	Calcium channel blockers, Dihydropyridines	mouse	Angiotensin II (Ang II)	5 and 20 mg/kg	everyday	gavage	yes	Treatment started before the initiation of AAA
Tomita 2008	C08CA05	Calcium channel blockers, Dihydropyridines	rat	porcine perfusion elastase (PPE)	10 mg/kg	everyday	Subcutaneous minipump	yes	Treatment started after the initiation of AAA
Yokokura 2007	C08CA	Calcium channel blockers, Dihydropyridines	rat	porcine perfusion elastase (PPE)	2 mg/kg	everyday	other	yes	Treatment started before the initiation of AAA
Mieth 2013	C08DB01	Calcium channel blockers, Non-dihydropyridines	mouse	Angiotensin II (Ang II)	100 mg/kg	everyday	with water	yes	Treatment started after the initiation of AAA
Wang 2018	C01AA05	Cardiac therapy,	mouse	porcine perfusion	2 mg/kg	everyday	gavage	yes	Treatment started before the initiation of AAA

		Cardiac glycosides		elastase (PPE)					
Wei 2014	C01AA05	Cardiac therapy, Cardiac glycosides	mouse	porcine perfusion elastase (PPE); Angiotensin II (Ang II)	20 µg/d and 40 µg/d	everyday	intraperitoneal injection	yes	Treatment started after the initiation of AAA
Ladd 2023	C03DA01	Diuretics, Aldosterone antagonists	mouse	porcine perfusion elastase (PPE)	1.5, 5, or 50 mg/kg	everyday	intraperitoneal injection	yes	Treatment started after the initiation of AAA
Wu 2022	C03XA01	Diuretics, Vasopressin antagonists	mouse	Angiotensin II (Ang II)	1 or 10 mg/kg	everyday	gavage	yes	Treatment started after the initiation of AAA
Amioka 2022	C10AB12	Lipid modifying agents, Fibrates	mouse	Angiotensin II (Ang II)	0.1 mg/kg	everyday	gavage	no	Treatment started before the initiation of AAA
Krishna 2012	C10AB05	Lipid modifying agents, Fibrates	mouse	Angiotensin II (Ang II)	100mg/kg	everyday	with water	yes	Treatment started before the initiation of AAA
Golledge 2010	C10AB05	Lipid modifying agents, Fibrates	mouse	Angiotensin II (Ang II)	Pioglitazone: 50 mg/kg, Fenofibrate: 100 mg/kg	everyday	with water	yes	Treatment started before the initiation of AAA
Horimatsu 2020	C10AD02	Lipid modifying agents, Nicotinic acid and derivatives	mouse	Angiotensin II (Ang II); calcium choride	(0.3% niacin) in drinking water	everyday	with water	yes	Treatment started before the initiation of AAA
Chen 2020	C10AX02	Lipid modifying	mouse	porcine perfusion	15 mg/kg , 60 mg/kg	everyday	gavage	yes	Treatment started after the initiation of AAA

		agents, Other lipid modifying agents		elastase (PPE)	and 130 mg/kg				
Yang 2019	C10AX02	Lipid modifying agents, Other lipid modifying agents	mouse	other	10 mg/kg or 20 mg/kg	everyday	Subcutaneous injection	yes	Treatment started after the initiation of AAA
Escudero 2015	C10AA07	Lipid modifying agents, Statins (HMG CoA reductase inhibitors)	mouse	Angiotensin II (Ang II)	bexarotene (10 mg/kg), rosuvastatin (10 mg/kg) or the combination of both	everyday	intramuscular injection; gavage	yes	Treatment started after the initiation of AAA
Golledge 2010	C10AA01	Lipid modifying agents, Statins (HMG CoA reductase inhibitors)	mouse	Angiotensin II (Ang II)	50 mg/kg	everyday	with water	yes	Treatment started before the initiation of AAA
Houdek 2013	C10AA05	Lipid modifying agents, Statins (HMG CoA reductase inhibitors)	other	porcine perfusion elastase (PPE)	1mg/kg	everyday	unspecified	no	Treatment started after the initiation of AAA
Wang 2011	C10AA07, C10AA05	Lipid modifying agents, Statins (HMG	mouse	Angiotensin II (Ang II)	rosuvastatin (10mg/kg/d) or	everyday	with water	no	Treatment started before the initiation of AAA

		CoA reductase inhibitors)			atorvastatin (20mg/kg/d)				
Kalyanasundaram 2006	C10AA01	Lipid modifying agents, Statins (HMG CoA reductase inhibitors)	rat	porcine perfusion elastase (PPE)	60 mg/kg	everyday	gavage	yes	Treatment started before the initiation of AAA
Kopacz 2020	C10AA01	Lipid modifying agents, Statins (HMG CoA reductase inhibitors)	mouse	Angiotensin II (Ang II)	20 mg/kg	everyday	gavage	yes	Treatment started before the initiation of AAA
Mastoraki 2012	C10AA01	Lipid modifying agents, Statins (HMG CoA reductase inhibitors)	rabbit	calcium choride	2 mg/kg	everyday	gavage	yes	Treatment started before the initiation of AAA
Meng 2022	C10AA01	Lipid modifying agents, Statins (HMG CoA reductase inhibitors)	mouse	Angiotensin II (Ang II)	50 mg/kg	everyday	gavage	yes	Treatment started before the initiation of AAA
Shiraya 2009	C10AA05	Lipid modifying agents, Statins (HMG	rat	porcine perfusion elastase (PPE)	20 mg/kg	everyday	gavage	yes	Treatment started after the initiation of AAA

		CoA reductase inhibitors)							
Steinmetz 2005	C10AA01	Lipid modifying agents, Statins (HMG CoA reductase inhibitors)	mouse	porcine perfusion elastase (PPE)	2 mg/kg	everyday	Subcutaneous injection	yes	Treatment started before the initiation of AAA
Takahashi 2013	C10AA05 and C08CA01	Lipid modifying agents, Statins (HMG CoA reductase inhibitors)	mouse	Angiotensin II (Ang II)	atorvastatin (10 mg/kg) and amlodipine (1 mg/kg)	everyday	gavage	yes	Treatment started before the initiation of AAA
Zhang 2009	C10AA01	Lipid modifying agents, Statins (HMG CoA reductase inhibitors)	mouse	Angiotensin II (Ang II)	10 mg/kg	everyday	Subcutaneous injection	yes	Treatment started before the initiation of AAA
Ma 2017	C10AA03	Lipid modifying agents, Statins (HMG CoA reductase inhibitors)	mouse	Angiotensin II (Ang II)	50 mg/kg	everyday	with water	yes	Treatment started after the initiation of AAA
Li 2017	C10AA05	Lipid modifying agents, Statins (HMG	mouse	Angiotensin II (Ang II); calcium choride	20mg/kg or 30mg/kg	everyday	gavage	yes	Treatment started after the initiation of AAA

		CoA reductase inhibitors)							
Nakahara 2024	C10AA05, C09CA01, C03DA01	Lipid modifying agents, Statins (HMG CoA reductase inhibitors) / Antihypertensives, Angiotensin II receptor blockers (ARBs) / Diuretics, Aldosterone antagonists	rat	calcium choride	20 mg/kg atorvastatin, 12.5 mg/kg losartan, 50 mg/kg aldactone, or 2.5 mg/kg risedronate	everyday	unspecified	yes	Treatment started before the initiation of AAA
Xiao 2020	D10AD02	Dermatologicals, Retinoids for acne treatment	mouse	Angiotensin II (Ang II)	5 mg/kg	everyday	gavage	yes	Treatment started before the initiation of AAA
Montgomery 2018	G04CA02	Genito-urinary system and sex hormones, Alpha-adrenoreceptor antagonists	mouse	other	0.125 µg/day or 0.250µg/day	everyday	Subcutaneous minipump	yes	Treatment started before the initiation of AAA
Wei 2024	G04CA03	Genito-urinary system and sex	mouse	Angiotensin II (Ang II); calcium choride	100 or 1000 mikrogram/kg	everyday	gavage	yes	Treatment started after the initiation of AAA

		hormones, Alpha-adrenoreceptor antagonists							
Davis 2016	G01AF11	Genito-urinary system and sex hormones, Anti-infectives and antiseptics	mouse	porcine perfusion elastase (PPE)	150 mg/kg	everyday	intraperitoneal injection	yes	Treatment started after the initiation of AAA
Yan 2020	G03XC02	Genito-urinary system and sex hormones, Sex hormones and modulators	mouse	Angiotensin II (Ang II)	5mg/kg/day	everyday	Subcutaneous injection	yes	Treatment started after the initiation of AAA
Dobrin 1996	H02AB04	Systemic hormonal preparations, Glucocorticoids	rat	porcine perfusion elastase (PPE)	1 mg/kg	everyday	Subcutaneous injection	yes	Treatment started after the initiation of AAA
Aoyama 2012	J01FA09	Antiinfectives for systemic use, Macrolides	mouse	calcium choride	100 mg/kg	everyday	with food	yes	Treatment started after the initiation of AAA
Kaito 2003	J01AA02	Antiinfectives for systemic use, Tetracyclines	rat	other	30 mg/kg per day	everyday	Subcutaneous injection	yes	Treatment started after the initiation of AAA
Manning 2003	J01AA02	Antiinfectives for systemic	mouse	Angiotensin II (Ang II)	30 mg/kg/d	everyday	with water	yes	Treatment started before the initiation of AAA

		use, Tetracyclines							
Mata 2015	J01AA02	Antiinfectives for systemic use, Tetracyclines	rat	other	30 mg/kg/day	everyday	gavage	yes	Treatment started before the initiation of AAA
Petrinec 1996	J01AA02	Antiinfectives for systemic use, Tetracyclines	rat	porcine perfusion elastase (PPE)	12.5 mg twice daily	everyday	Subcutaneous injection	yes	Treatment started after the initiation of AAA
Prall 2002	J01AA02	Antiinfectives for systemic use, Tetracyclines	mouse	calcium choride	10, 50, and 100 mg/kg	everyday	with water	yes	Treatment started after the initiation of AAA
Xie 2012	J01AA02	Antiinfectives for systemic use, Tetracyclines	mouse	Angiotensin II (Ang II)	100 mg/kg/day	everyday	with water	no	Treatment started after the initiation of AAA
Yu 2017	J01AA02	Antiinfectives for systemic use, Tetracyclines	mouse	porcine perfusion elastase (PPE)	gavage p.o. 30 mg/kg of DOX every day or every other day and intraperitoneal injection of 15 mg/kg of DOX every other day	everyday; every 2 days	gavage; intraperitoneal injection	yes; no	Treatment started after the initiation of AAA
Sho 2004	J01AA02	Antiinfectives for systemic use, Tetracyclines	rat	porcine perfusion elastase (PPE)	PDS 1.5 mg/kg/day, SC 30 mg/kg/day	everyday	periaortic delivery system, Subcutaneous injection	yes	Treatment started after the initiation of AAA

Curci 1998	J01AA02	Antiinfectives for systemic use, Tetracyclines	rat	porcine perfusion elastase (PPE)	7.5 mg/kg/day, 15 mg/kg/day, 30 mg/kg/day, 60 mg/kg/day	everyday	Subcutaneous injection	yes	Treatment started after the initiation of AAA
Adams 2021	J01AA02	Antiinfectives for systemic use, Tetracyclines	mouse	Angiotensin II (Ang II)	30 mg/kg	everyday	Subcutaneous minipump	no	Treatment started after the initiation of AAA
Culpan 2022	J01MA02	Other / Unclassified	rat	calcium choride	100 mg	everyday	intraperitoneal injection	yes	Treatment started after the initiation of AAA
LeMaire 2018	J01MA02	Other / Unclassified	mouse	Angiotensin II (Ang II)	100 mg/kg/d	everyday	gavage	yes	Treatment started after the initiation of AAA
Iida 2012	J01AA02	Other / Unclassified / Antiinfectives for systemic use, Tetracyclines	mouse	porcine perfusion elastase (PPE); Angiotensin II (Ang II)	Irbesartan (50 mg/kg), telmisartan (10 mg/kg), fluvastatin (40 mg/kg), bosentan (100 mg/kg), doxycycline (100 mg/kg)	everyday	with food; with water	no	Treatment started before the initiation of AAA
Busch 2021	L01EX08	Antineoplastic and immunomodulating agents, Antineoplastic agents	mouse	porcine perfusion elastase (PPE); Angiotensin II (Ang II)	(5 mg/kg) of 2.5 mg/mL lenvatinib	everyday	gavage	yes	Treatment started after the initiation of AAA
Escudero 2015	L01XF03	Antineoplastic and immunomodu	mouse	Angiotensin II (Ang II)	bexarotene (10 mg·kg−1·day	everyday	intramuscular injection; gavage	yes	Treatment started after the initiation of AAA

		lating agents, Antineoplastic agents			−1), rosuvastatin (10 mg·kg−1·day −1) or the combination of both				
Ghoshal 2012	L01XX33	Antineoplastic and immunomodulating agents, Antineoplastic agents	mouse	Angiotensin II (Ang II)	1000 ppm	everyday	with food	yes	Treatment started after the initiation of AAA
Lawrence 2004	L01EG04	Antineoplastic and immunomodulating agents, Antineoplastic agents	rat	porcine perfusion elastase (PPE)	1 mg/kg/d	everyday	gavage	yes	Treatment started after the initiation of AAA
Li 2018	L01EG04	Antineoplastic and immunomodulating agents, Antineoplastic agents	mouse	Angiotensin II (Ang II)	2mg/kg/d	everyday	intraperitoneal injection	yes	Treatment started after the initiation of AAA
Moran 2013	L01EG02	Antineoplastic and immunomodulating agents, Antineoplastic agents	mouse	Angiotensin II (Ang II)	1.04 μg/kg/min	everyday	Subcutaneous minipump	yes	Treatment started after the initiation of AAA
Obama 2015	L01EB02	Antineoplastic and immunomodulating agents,	mouse	Angiotensin II (Ang II)	7.5 mg/kg/day	everyday	Subcutaneous minipump	yes	Treatment started after the initiation of AAA

		Antineoplastic agents							
Ren 2015	L01XG01	Antineoplastic and immunomodulating agents, Antineoplastic agents	mouse	Angiotensin II (Ang II)	50μg/kg, 2 times per week	other	intraperitoneal injection	yes	Treatment started after the initiation of AAA
Vorkapic 2016	L01EA01	Antineoplastic and immunomodulating agents, Antineoplastic agents	mouse	Angiotensin II (Ang II)	10 mg/kg	everyday	gavage	yes	Treatment started before the initiation of AAA
Yao 2019	L01EA01	Antineoplastic and immunomodulating agents, Antineoplastic agents	rat	porcine perfusion elastase (PPE)	50 mg/kg/d	everyday	gavage	yes	Treatment started after the initiation of AAA
Davis 2016	L02BB01	Antineoplastic and immunomodulating agents, Endocrine therapy	mouse	porcine perfusion elastase (PPE)	flutamide (50 mg/kg) or ketoconazole (150 mg/kg)	everyday	intraperitoneal injection	yes	Treatment started after the initiation of AAA
Grigoryants 2005	L02BA01	Antineoplastic and immunomodulating agents, Endocrine therapy	rat	porcine perfusion elastase (PPE)	10 mg/kg	everyday	Subcutaneous injection	yes	Treatment started before the initiation of AAA
Johnston 2013	L04AC03	Antineoplastic and immunomodu	mouse	porcine perfusion	10 mg/kg per day, 30 mg/kg per	everyday	Subcutaneous minipump	yes	Treatment started after the initiation of AAA

		lating agents, Immunosuppressants		elastase (PPE)	day or 100 mg/kg per day				
Li 2017	L04AH01	Antineoplastic and immunomodulating agents, Immunosuppressants	mouse	porcine perfusion elastase (PPE)	2 mg/kg	everyday	intraperitoneal injection	yes	Treatment started after the initiation of AAA
Rouer 2014	L04AH01	Antineoplastic and immunomodulating agents, Immunosuppressants	mouse	porcine perfusion elastase (PPE)	5 mg/kg/day	everyday	gavage	yes	Treatment started after the initiation of AAA
Millar 2024	L04AC03	Antineoplastic and immunomodulating agents, Immunosuppressants	mouse	porcine perfusion elastase (PPE)	100 mg/kg	everyday	intraperitoneal injection	yes	Treatment started after the initiation of AAA
Yong 2023	L04AX05	Antineoplastic and immunomodulating agents, Immunosuppressants	mouse	Angiotensin II (Ang II)	200 mg/kg/day	everyday	gavage	yes	Treatment started after the initiation of AAA
Song 2024	L04AX04	Antineoplastic and immunomodulating agents, Immunosuppressants	mouse	Angiotensin II (Ang II)	20 mg/kg/day	everyday	gavage	yes	Treatment started before the initiation of AAA
Wang 2025	L04AX07	Antineoplastic and	mouse	porcine perfusion	100 mg/kg/d	everyday	gavage	yes	Treatment started after the initiation of AAA

		immunomodulating agents, Immunosuppressants		elastase (PPE); calcium choride					
Dobrin 1996	L04AD01	Antineoplastic and immunomodulating agents, Immunosuppressants	rat	porcine perfusion elastase (PPE)	1 mg/kg per day of methylprednisolone or 5 mg/kg of cyclosporine twice per day beginning i	everyday	unspecified	yes	Treatment started after the initiation of AAA
Marinkovic 2013	L04AX01	Antineoplastic and immunomodulating agents, Immunosuppressants	mouse	Angiotensin II (Ang II)	0.1 mg/kg/day	everyday	with food	yes	Treatment started after the initiation of AAA
Griepke 2022	L04AB01	Antineoplastic and immunomodulating agents, Immunosuppressants	mouse	porcine perfusion elastase (PPE)	20 mg/kg	every 3 days	intraperitoneal injection	yes	Treatment started after the initiation of AAA
Phie 2022	M04AC01	Musculoskeletal system, Antigout preparations	mouse	other	0.2 mg/kg/d	everyday	gavage	no	Treatment started after the initiation of AAA
Zhao 2023	M04AC01	Musculoskeletal system, Antigout preparations	mouse	porcine perfusion elastase (PPE)	0.1 mg/kg/day	everyday	intraperitoneal injection	yes	Treatment started after the initiation of AAA
Hu 2024	M04AC01	Musculoskeletal system,	mouse	Angiotensin II (Ang II);	0.4 mg/kg	everyday	gavage	yes	Treatment started after the initiation of AAA

		Antigout preparations		calcium choride					
Chen 2024	M04AC01	Musculoskeletal system, Antigout preparations	mouse	Angiotensin II (Ang II); calcium choride	0.2 mg/kg/day	everyday	gavage	yes	Treatment started after the initiation of AAA
Tsai 2013	M05BA08	Musculoskeletal system, Bisphosphonates	mouse	Angiotensin II (Ang II)	100 μg/kg	everyday	intraperitoneal injection	yes	Treatment started after the initiation of AAA
Nakahara 2024	M05BA07	Musculoskeletal system, Bisphosphonates	rat	calcium choride	2.5 mg/kg	everyday	unspecified	yes	Treatment started before the initiation of AAA
King 2006	M01AH01	Musculoskeletal system, COX-2 inhibitors	mouse	Angiotensin II (Ang II)	125 mg /kg/ day	everyday	with food	yes	Treatment started before the initiation of AAA
Holmes 1996	M01AB01	Musculoskeletal system, Non-steroidal anti-inflammatory drugs (NSAIDs), Acetic acid derivatives	rat	porcine perfusion elastase (PPE)	1.6 mg/day	everyday	Subcutaneous injection	yes	Treatment started after the initiation of AAA
Miralles 1999	M01AB01	Musculoskeletal system, Non-steroidal anti-inflammatory drugs (NSAIDs),	rat	porcine perfusion elastase (PPE)	1.6 mg/kg	everyday	Subcutaneous injection	yes	Treatment started after the initiation of AAA

		Acetic acid derivatives							
Chen 2020	N03AX11	Nervous system, Antiepileptics	mouse	Angiotensin II (Ang II)	3 mg/day and 6 mg/day	everyday	intraperitoneal injection	yes	Treatment started after the initiation of AAA
Zhu 2024	N03AX11	Nervous system, Antiepileptics	rat	porcine perfusion elastase (PPE); calcium choride	100 mg/kg/d	everyday	gavage	yes	Treatment started after the initiation of AAA
Guo 2023	N07BB01	Nervous system, Drugs used in addictive disorders	mouse	Angiotensin II (Ang II)	50 mg/kg/day	everyday	gavage	yes	Treatment started before the initiation of AAA
Kong 2017	N05CH01	Nervous system, Hypnotics and sedatives	mouse	Angiotensin II (Ang II)	30mg/ kg/ day	everyday	intraperitoneal injection	yes	Treatment started after the initiation of AAA
Tang 2017	N05CH01	Nervous system, Hypnotics and sedatives	rat	porcine perfusion elastase (PPE)	10 mg/kg/d	everyday	gavage; intraperitoneal injection	yes	Treatment started before the initiation of AAA
Yu 2023	N05CM18	Nervous system, Hypnotics and sedatives	rat	porcine perfusion elastase (PPE)	50 μg/kg/day	everyday	intraperitoneal injection	yes	Treatment started after the initiation of AAA
Tekin 2018	N05CH01	Nervous system, Hypnotics and sedatives	rat	calcium choride	10 mg/kg/day	everyday	intraperitoneal injection	yes	Treatment started after the initiation of AAA
Yu 2021	N05CM18	Nervous system,	rat	porcine perfusion elastase	50 μg/kg	everyday	intraperitoneal injection	yes	Treatment started after the initiation of AAA

		Hypnotics and sedatives		(PPE); calcium choride					
Yang 2025	N06BX18	Nervous system, Psychoanaleptics	mouse	porcine perfusion elastase (PPE)	5 mg/kg	everyday	intraperitoneal injection	yes	Treatment started before the initiation of AAA
Uchida 2022	N07XX14	Other / Unclassified	mouse	Angiotensin II (Ang II)	10 mg/kg/day	everyday	intraperitoneal injection	yes	Treatment started before the initiation of AAA
Morimoto 2012	N07XX14	Other / Unclassified	rat	porcine perfusion elastase (PPE); calcium choride	1 mg/kg/d or 5 mg/kg/d twice daily	everyday	intraperitoneal injection	yes	Treatment started before the initiation of AAA
Ramadan 2015	P01BA01	Antiparasitic products, Antiprotozoals	mouse	Angiotensin II (Ang II)	50mg/kg	everyday	intraperitoneal injection	no	Treatment started after the initiation of AAA
Liao 2022	P03AA04	Antiparasitic products, Ectoparasiticides including scabicides	mouse	Angiotensin II (Ang II)	50 mg/kg	everyday	gavage	yes	Treatment started after the initiation of AAA
DiGennaro 2018	R03DC03	Respiratory system, Leukotriene receptor antagonists	mouse	calcium choride	montelukast 0.1 mg/kg/d and montelukast 1 mg/kg/d	everyday	unspecified	yes	Treatment started after the initiation of AAA
Kawai 2019	R03DC03	Respiratory system, Leukotriene receptor antagonists	mouse	Angiotensin II (Ang II)	10 mg/kg/day	everyday	gavage	yes	Treatment started after the initiation of AAA

Huang 2024	R03DC03	Respiratory system, Leukotriene receptor antagonists	mouse	Angiotensin II (Ang II)	10 mg/kg	everyday	gavage	yes	Treatment started after the initiation of AAA
Li 2024	R03DC03	Respiratory system, Leukotriene receptor antagonists	mouse	porcine perfusion elastase (PPE)	1 or 10 mg/kg, daily	everyday	gavage	yes	Treatment started before the initiation of AAA
Zhang 2024	R03AC13	Respiratory system, Selective beta-2-adrenoreceptor agonists	mouse	other	0.3 mg/kg/day or 1mg/ kg/ day or 3mg/kg/day	everyday	intraperitoneal injection	yes	Treatment started after the initiation of AAA
Xie 2024	S01LA01	Sensory organs, Antineovascularisation agents	mouse	porcine perfusion elastase (PPE); calcium choride	30 µg	every 2 days	intraperitoneal injection	yes	Treatment started before the initiation of AAA

Table S2. Risk of bias table.

Study ID	ATC Code	Selection Bias	Performance Bias	Detection Bias	Attrition Bias	Reporting Bias	Other Bias	Overall Assessment
Shiraya 2009	C10AA05	Low	Low	Low	Low	Low	Low	Low
Chen 2020	N03AX11	Low	Unclear	Low	Low	Low	Low	Low
Phie 2022	M04AC01	Low	Low	Low	Unclear	Low	Low	Low
Tang 2017	N05CH01	Low	Unclear	Low	Low	Low	Low	Low
Li 2017	L04AH01	Low	Unclear	Low	Low	Low	Low	Low
Krishna 2012	C10AB05	Low	Low	Low	Low	Low	Low	Low
Kalyanasundaram 2006	C10AA01	Low	Low	Low	Low	Low	Low	Low
Houdek 2013	C10AA05	Unclear	Unclear	Unclear	Unclear	Unclear	Unclear	Medium
Xie 2012	J01AA02	Unclear	High	High	Low	Low	Low	Medium
Li 2024	R03DC03	Low	Unclear	Unclear	Low	Low	Unclear	Medium
Alsac 2011	C09AA04	Low	Unclear	Unclear	Low	Low	Unclear	Medium
LeMaire 2018	J01MA02	Low	Unclear	Unclear	Low	Low	Unclear	Medium
Obama 2015	L01EB02	Unclear	Unclear	Unclear	Low	Low	Unclear	Medium
Li 2020	B02AB05	Unclear	Unclear	Low	Low	Low	Low	Medium
Ghoshal 2012	L01XX33	Unclear	High	High	Low	Low	Unclear	Medium
Hu 2024	M04AC01	Unclear	Low	Low	Low	Low	Unclear	Medium
Guo 2023	N07BB01	Unclear	Low	Low	Low	Low	Unclear	Medium
Zhao 2024	A10BJ02	Unclear	High	High	Low	Low	Unclear	Medium
Kunath 2021	A10BA02	Low	Unclear	Unclear	Low	Low	Low	Medium
Holmes 1996	M01AB01	Unclear	High	High	Low	Low	Unclear	Medium
Mata 2015	J01AA02	Unclear	High	High	Low	Low	Unclear	Medium
Sho 2004	J01AA02	Unclear	High	Low	Low	Low	Low	Medium
Wei 2024	G04CA03	Low	Unclear	Unclear	Low	Low	Unclear	Medium
Rouer 2014	L04AH01	Unclear	High	Low	Low	Low	Low	Medium

Bao 2014	A10BH04	Low	Unclear	Unclear	Low	Low	Unclear	Medium
Yu 2016	A10BJ03	Unclear	High	High	Low	Low	Unclear	Medium
Park 2018	C09AA05, C07AG02	Low	Unclear	Unclear	Low	Low	Unclear	Medium
Li 2017	L04AH01	Low	Unclear	Unclear	Low	Low	Low	Medium
Grigoryants 2005	L02BA01	Low	Unclear	Unclear	Low	Low	Low	Medium
Millar 2024	L04AC03	Unclear	High	High	Low	Low	Unclear	Medium
Ren 2015	L01XG01	Unclear	High	High	Low	Low	Unclear	Medium
Yan 2020	G03XC02	Low	Unclear	Unclear	Low	Low	Low	Medium
Moran 2013	L01EG02	Unclear	High	High	Low	Low	Unclear	Medium
Steinmetz 2005	C10AA01	Unclear	High	High	Low	Low	Unclear	Medium
Wang 2023	C02DB02	Low	Unclear	Unclear	Low	Low	Unclear	Medium
Owens 2015	B01AC06 or B01AC04	Unclear	High	High	Low	Low	Low	Medium
Huang 2024	R03DC03	Low	Unclear	Unclear	Low	Low	Unclear	Medium
Golledge 2010	C10AA01	Unclear	High	High	Low	Low	Low	Medium
Xu 2023	A10BA02	Unclear	High	Low	Low	Low	Low	Medium
Hu 2024	S01LA01	Unclear	Low	Low	Low	Unclear	Unclear	Medium
Culpan 2022	J01MA02	Low	Unclear	Low	Low	Low	Unclear	Medium
Fujiwara 2008	C09CA03	Unclear	High	High	Low	Low	Unclear	Medium
Wang 2011	C10AA07 or C10AA05	Low	Unclear	Low	Low	Low	Unclear	Medium
Lawrence 2004	L01EG04	Low	Unclear	Unclear	Low	Low	Low	Medium
Ortega 2019	A10BK03	Low	Unclear	Low	Low	Low	Unclear	Medium
Manning 2003	J01AA02	Unclear	High	High	Low	Low	Unclear	Medium
Liao 2022	P03AA04	Low	Unclear	Low	Low	Low	Unclear	Medium
Montgomery 2018	G04CA02	Unclear	High	High	Low	Low	Unclear	Medium
Tekin 2018	N05CH01	Low	Unclear	Unclear	Low	Low	Unclear	Medium

Moran 2017	B01AB05, B01AX05 or B01AE07	Low	Unclear	Unclear	Low	Low	Unclear	Medium
Morimoto 2012	N07XX14	Unclear	High	High	Low	Low	Unclear	Medium
Fu 2011	C09AA07	Unclear	High	High	Low	Low	Unclear	Medium
Zhang 2011	B01AC23	Low	Unclear	Low	Low	Low	Unclear	Medium
Zhu 2024	N03AX11	Low	Unclear	Unclear	Low	Low	Unclear	Medium
Kaschina 2008	C09CA07 or C02DB02	Low	Unclear	Unclear	Low	Low	Unclear	Medium
Jones 2009	A10BG02	Unclear	High	High	Low	Low	Unclear	Medium
Wu 2022	C03XA01	Unclear	High	High	Low	Low	Unclear	Medium
Seto 2014	C09XA02	Low	Unclear	Unclear	Low	Low	Unclear	Medium
Hao 2018	C09AA	Low	Unclear	Unclear	Low	Low	Unclear	Medium
Mastoraki 2012	C10AA01	Low	Unclear	Unclear	Low	Low	Unclear	Medium
Xiao 2020	D10AD02	Low	Unclear	Unclear	Low	Low	Unclear	Medium
He 2021	A10BA02	Low	Unclear	Low	Low	Low	Unclear	Medium
Yu 2021	N05CM18	Unclear	High	High	Low	Low	Low	Medium
Kawai 2019	R03DC03	Unclear	High	High	Low	Low	Unclear	Medium
Adams 2021	J01AA02	Unclear	High	High	Low	Low	Unclear	Medium
Amioka 2022	C10AB12	Unclear	High	High	Low	Low	Unclear	Medium
Liu 2022	A10BK01	Unclear	High	High	Low	Low	Unclear	Medium
Meng 2022	C10AA01	Low	Unclear	Unclear	Low	Low	Unclear	Medium
Dobrin 1996	H02AB04 or L04AD01	Unclear	High	Low	Low	Low	Unclear	Medium
Takahashi 2012	C10AA05 and C08CA01	Low	Unclear	Unclear	Low	Low	Unclear	Medium
Xiong 2014	C09AA04	Low	Unclear	Unclear	Low	Low	Low	Medium
Nakahara 2024	C10AA05, C09CA01, C03DA01 or M05BA07	Low	Unclear	Unclear	Low	Low	Unclear	Medium
Petrinec 1996	J01AA02	Unclear	Unclear	Unclear	Low	Low	Unclear	Medium

Curci 1998	J01AA02	Unclear	Low	Low	Low	Unclear	Unclear	Medium
Iida 2012	C09CA04, C09CA07, C10AA04, C02KX01 or J01AA02	Unclear	High	High	Low	Low	High	High
Mieth 2013	C08DB01	Unclear	High	High	Low	Low	Unclear	High
Yokokura 2007	C08CA	Unclear	High	High	Low	Low	Unclear	High
Zhang 2009	C10AA01	Unclear	High	High	Low	Low	Unclear	High
Ma 2017	C10AA03	High	High	High	Unclear	High	Unclear	High
Wei 2014	C01AA05	Unclear	High	High	Low	Low	Unclear	High
Takahara 2018	A10BH08	Unclear	High	High	Low	Low	Unclear	High
DiGennaro 2018	R03DC03	Unclear	High	High	Low	Low	Unclear	High
Hall 2021	C09CA01	Unclear	High	High	Low	Low	Unclear	High
King 2006	M01AH01	Unclear	High	High	Low	Low	Unclear	High
Tsai 2013	M05BA08	High	High	High	Unclear	Low	Unclear	High
Aoyama 2012	J01FA09	Unclear	High	High	Unclear	Low	Unclear	High
Yao 2019	L01EA01	Unclear	High	High	Low	Low	Unclear	High
Miralles 1999	M01AB01	High	High	High	High	High	High	High
Wang 2019	A10BA02	Unclear	High	High	Low	Low	Unclear	High
Umebayashi 2018	B01AC23	Unclear	High	High	Low	Low	Unclear	High
Kohashi 2016	A10BJ	Unclear	High	High	Low	Low	High	High
Miyake 2017	C09XA02	Unclear	High	High	Low	Low	Unclear	High
Wang 2025	L04AX07	Unclear	High	High	Low	Low	Unclear	High
Slaiby 1994	C07AA05	Unclear	High	High	Low	Low	Unclear	High
Escudero 2015	L01XF03 and C10AA07	Unclear	High	High	Low	Low	Unclear	High
Inoue 2009	C09CA06, C09AA03	Unclear	High	High	Low	Low	Unclear	High
Horimatsu 2020	C10AD02	High	High	High	Unclear	Low	Low	High
Busch 2021	L01EX08	Unclear	High	High	Low	Low	Low	High

Uchida 2022	N07XX14	Unclear	High	High	Low	Low	Unclear	High
Ding 2021	B01AF01	Unclear	High	High	Low	Low	Unclear	High
NguyenTLL 2024	C09CA01	Unclear	High	High	Low	Low	Unclear	High
Jreije 2022	A11CC04	Unclear	High	High	Low	Low	Unclear	High
Liu 2012	B01AC04	Unclear	High	High	Low	Low	Low	High
Lu 2015	A10BH01	Unclear	High	High	Low	Low	Unclear	High
Daugherty 2001	C09CA01	High	High	High	High	High	High	High
Yu 2023	N05CM18	Unclear	High	High	Low	Low	Low	High
Tomita 2008	C08CA05	Unclear	High	High	Low	Low	Unclear	High
Hosokawa 2010	C09CA08	Unclear	High	High	Low	Low	Unclear	High
Liao 2001	C09AA01, C09AA03, C09AA02, C09CA01	Unclear	High	High	Low	Low	Low	High
Martorell 2016	A11CC04	Unclear	High	High	Low	Low	Unclear	High
Miao 2013	C08CA05	Unclear	High	High	Low	Low	Low	High
Zhao 2023	M04AC01	Unclear	High	High	Low	Low	Low	High
Vorkapic 2016	L01EA01	Unclear	High	High	Low	Low	Low	High
Wang 2018	A10BG02	Unclear	High	High	Low	Low	Unclear	High
Kopacz 2020	C10AA01	Unclear	High	High	Low	Low	Unclear	High
Ramadan 2015	P01BA01	Unclear	High	High	Low	Low	Low	High
Golledge 2010	C10AA01	Unclear	High	High	Low	Low	Unclear	High
Li 2018	L01EG04	Unclear	High	High	Unclear	Low	Low	High
Simpson 1970	C07AA05	Unclear	High	High	Low	Low	Low	High
Chen 2024	M04AC01	High	High	High	Unclear	Low	Unclear	High
Ricci 1996	C07AA05	Unclear	High	High	Low	Low	Unclear	High
Griepke 2022	L04AB01	Unclear	High	High	Low	Low	Low	High
Huang 2022	B03BB01+C08CA05	High	High	High	Unclear	High	High	High
Chen 2013	C08CA01	Unclear	High	High	High	Low	Unclear	High
Yang 2025	N06BX18	Unclear	High	High	Low	Low	Unclear	High

Marinkovic 2013	L04AX01	Unclear	High	High	Low	Low	Unclear	High
Yu 2017	J01AA02	High	High	High	High	High	High	High
Takai 2013	C09AA16	Unclear	High	High	Low	Low	Unclear	High
Wang 2018	A10BG02	Unclear	High	High	Low	Low	Unclear	High
Yong 2023	L04AX05	Unclear	High	High	Unclear	Low	Unclear	High
Yang 2019	C10AX02	Unclear	High	High	Low	Low	Unclear	High
Kong 2017	N05CH01	Unclear	High	High	Low	Low	Unclear	High
Davis 2016	L02BB01, G01AF11	Unclear	High	High	Low	Low	Unclear	High
Johnston 2013	L04AC03	Unclear	High	High	Low	Low	Unclear	High
AlSiraj 2021	C09CA03, C09DX04	Unclear	High	High	Unclear	Low	Low	High
Song 2024	L04AX04	Unclear	High	High	Low	Low	Unclear	High
Zhang 2024	R03AC13	Unclear	High	High	Low	Low	Unclear	High
Karasaki 2023	C09CA08	High	High	High	High	High	High	High
Kaito 2003	J01AA02	Unclear	High	High	Low	Low	Unclear	High
Chen 2020	N03AX11	High	High	High	Unclear	Low	Unclear	High
Ladd 2023	C03DA01	Unclear	High	High	Low	Low	Unclear	High
Xuan 2018	C09CA07	Unclear	High	High	Unclear	Low	Unclear	High
Prall 2002	J01AA02	Unclear	High	High	Low	Low	Unclear	High
